# Use of other antimicrobial drugs is associated with trimethoprim resistance in patients with urinary tract infections caused by *E. coli*

**DOI:** 10.1007/s10096-019-03672-2

**Published:** 2019-09-07

**Authors:** M. Mulder, A. Verbon, J. Lous, W. Goessens, B. H. Stricker

**Affiliations:** 1 Department of Epidemiology, Erasmus Medical Center, Erasmus University, PO Box 2040, 3000 CA Rotterdam, The Netherlands; 2 Inspectorate for Healthcare and Youth, PO Box 2518, 6401 DA Heerlen, The Netherlands; 3 Department of Medical Microbiology and Infectious Diseases, Erasmus Medical Center, Erasmus University, PO Box 2040, 3000 CA Rotterdam, The Netherlands; 4 Star-SHL, PO Box 8661, 3009 AR Rotterdam, The Netherlands; 5 Department of Internal Medicine, Erasmus Medical Center, Erasmus University, PO Box 2040, 3000 CA Rotterdam, The Netherlands

**Keywords:** Trimethoprim resistance, UTIs, *E. coli*, Antimicrobial drugs, Sulfonamides and trimethoprim, Beta-lactam antibacterials, Nitrofuran derivatives

## Abstract

In recent years, high frequencies of trimethoprim resistance in urinary tract infections (UTIs) caused by *E. coli* are have been reported. Co-resistance to other antimicrobial drugs may play a role in this increase. Therefore, we investigated whether previous use of other antimicrobial drugs was associated with trimethoprim resistance. We conducted a nested case-control study with urinary cultures with *E. coli* from participants of the Rotterdam Study sent in by general practitioners to the regional laboratory between 1 January 2000 and 1 April 2016. Multivariable logistic regression analysis was performed to study the association between prior prescriptions of several antimicrobial drug groups and trimethoprim resistance using individual participant data. Urinary cultures of 1264 individuals with a UTI caused by *E. coli* were included. When adjusted for previous other antimicrobial drug use, a history of > 3 prescriptions of extended-spectrum penicillins (OR 1.68; 95% CI 1.10–2.55) was significantly associated with trimethoprim resistance of *E. coli* as was the use of > 3 prescriptions of sulfonamides and trimethoprim (OR 2.22; 95% CI 1.51–3.26). The use of > 3 prescriptions of nitrofuran derivatives was associated with a lower frequency of trimethoprim resistance (OR 0.60; 95% CI 0.39–0.92), after adjustment for other antimicrobial drug prescriptions. We found that previous use of extended-spectrum penicillins is associated with trimethoprim resistance. On the contrary, previous nitrofurantoin use was associated with a lower frequency of trimethoprim resistance. Especially in individuals with recurrent UTI, co-resistance should be taken into account and susceptibility testing before starting trimethoprim should be considered.

**Electronic supplementary material:**

The online version of this article (10.1007/s10096-019-03672-2) contains supplementary material, which is available to authorized users.

## Introduction

The increase in antimicrobial resistance is becoming a threat to the treatment of infections and is directly associated with the increasing use of antimicrobial drugs [1]. Antimicrobial drugs are frequently prescribed for urinary tract infections (UTIs) by general practitioners (GPs). Trimethoprim has often been prescribed, especially in women with UTIs, and high frequencies of trimethoprim resistance have been reported [2]. Urinary cultures from female students from the USA, positive for *E. coli* of 2005–2007, were in 29.6% of the cases resistant to trimethoprim. In the UK, there was 29% resistance in community UTIs in 2015 and 34% in 2016. In the Netherlands, the resistance rates for trimethoprim in outpatient UTIs caused by *E. coli* increased from 15% in 2000 to 31% in 2010 [3–5]. Use of trimethoprim or other antibiotics was shown to be an important risk factor [6]. Co-resistance, the simultaneous resistance of one microbe for two or more antimicrobial drugs, has been suggested to play a role [4]. For example, co-resistance for trimethoprim and amoxicillin means that use of amoxicillin not only selects for amoxicillin-resistant but also for trimethoprim-resistant microorganisms. One of the mechanisms may be that resistance genes are present on the same plasmid [7].

That co-resistance may be of importance was described in a recent study in the UK, which showed an association between higher prescribing rates of extended-spectrum penicillins (such as amoxicillin) and trimethoprim resistance in *Enterobacteriaceae* causing UTIs. Interestingly, an association with reduced trimethoprim resistance was shown for nitrofurantoin and macrolide use [8]. These associations were at population level and not based on data from individual patients.

In recent years, trimethoprim has been replaced by nitrofurantoin in primary care guidelines in the UK and the Netherlands [4, 5, 9, 10]. Although, trimethoprim resistance rates substantially decreased with the changing guidelines, they remain high with 24% resistance in *E. coli* causing UTIs in primary care patients in the Netherlands in 2017 [11]. With the unique opportunity of urinary cultures and antimicrobial drug prescriptions at the individual level from participants of the prospective population-based Rotterdam Study, we were able to study the effects of prescriptions of several antimicrobial drug groups on trimethoprim resistance.

## Materials and methods

### Source population

We conducted a nested case-control study, using urinary cultures obtained from participants of the Rotterdam Study, a prospective cohort study in older adults in the Ommoord area, Rotterdam, The Netherlands. The Rotterdam Study was described elsewhere [12]. In short, the study started in 1991, when all inhabitants of Ommoord aged ≥ 55 years were invited to participate (78% response rate). New cohorts of inhabitants of ≥ 45 years were included later, resulting in 14,926 participants in three cohorts. All participants are invited every few years for interviews and examinations. The Rotterdam Study has been approved by the Medical Ethics Committee of the Erasmus MC and by the Ministry of Health, Welfare and Sport of the Netherlands, implementing the Wet Bevolkingsonderzoek: ERGO (Population Studies Act: Rotterdam Study). All participants provided written informed consent to participate in the study and to obtain information from their treating physicians*.*

### Study population

The study population included all participants of the Rotterdam Study of whom at least one urinary culture was assessed at the Star-SHL laboratory between January 1, 2000, and April 12, 2016, and which was positive (at least 10^3^ cfu/mL) for *E. coli*. The Star-SHL laboratory provides laboratory services for all GPs in the Rotterdam area, including Ommoord. Midstream urine was collected and sent to the Star-SHL laboratory according to the national guidelines of routine care for GPs in The Netherlands. These guidelines recommend culturing only when the patient has clinical signs of a UTI, belongs to a risk group, suffers from a complicated infection, or has complaints that did not disappear despite empiric treatment. When multiple cultures of one individual were sent in, only the first one was used.

### Cases and controls

Cases were individuals with a first urinary culture positive for *E. coli* resistant to trimethoprim, whereas controls were individuals with a first urinary culture positive for *E. coli* susceptible to trimethoprim in the study period. Before 2010, disk diffusion was used to determine the susceptibility of the *E. coli* to trimethoprim, whereas after 2010, susceptibility testing was performed with the VITEK 2 system (VITEK AMS; bioMerieux Vitek systems, Inc., Hazelwood, MO, USA). In order to use the same cut-off points, all MIC-values were interpreted according to the CSLI criteria of before 2010. Intermediate susceptibility was considered resistant in the analysis, resulting in a cut-off of > 4 μg/mL. During the period of disk diffusion susceptibility testing (before 2010), the MIC breakpoint of trimethoprim has changed from > 8 to > 4 μg/mL. This may have led to misclassification in this period, but since only 2 out of 586 isolates had a MIC of 4 μg/mL, it can safely be assumed that only a very small proportion of the cultures could have been misclassified. Furthermore, susceptibility of the *E. coli* for amoxicillin (> 8 μg/mL), amoxicillin-clavulanic acid (> 8/4 μg/mL), nitrofurantoin (> 32 μg/mL), and ciprofloxacin (> 4 μg/mL) were determined.

### Use of antimicrobial drugs

Computerized records from the pharmacies in the Ommoord district were used to study medication use of the participants. The total number of prescriptions of all antimicrobial drugs between January 1, 1995, and the date of the culture was determined. All generations of cephalosporins were excluded because they were only prescribed to 3 individuals in the study period. This resulted in the following antimicrobial drug groups: ATC-codes J01AA (tetracyclines), J01CA (extended-spectrum penicillins), J01CR (combinations of penicillins incl. beta-lactamase inhibitors, J01E (sulfonamides and trimethoprim), J01F (macrolides and lincosamides), J01MA (fluoroquinolones), J01XE (nitrofuran derivatives). For each antimicrobial drug group, exposure per individual was categorized according to the number of prescriptions during the study period: 0, 1, 2 or 3, or > 3 prescriptions.

Furthermore, for all groups, the date of the last prescription before culture was obtained, after which the time interval between the antimicrobial drug prescription and urinary culture was calculated. These time intervals were categorized into use 1–3 months, 3–12 months, and > 12 months before urinary culture and compared with no use at all during the study period. Prescriptions in the month before culture were excluded from the analysis, since it could not be excluded that these antimicrobial drugs were prescribed for the UTI that led to the culture.

### Confounders

All analyses were adjusted for sex and age. Serum creatinine was used to calculate the glomerular filtration rate (GFR), according to the CKD-EPI equation, using the value closest to culture [13]. Diabetes mellitus was defined as use of anti-diabetic medication at the moment of culture. Socioeconomic status (SES) was scored at baseline according to the UNESCO criteria. To account for potential bias associated with missing data, missing values on kidney function (18.4%) and SES (1.3%) were imputed using multiple imputation (*N* = 20 imputations) in SPSS using the default settings [14]. All available variables (sex, age, diabetes, the use of the different antimicrobial drugs, and the different resistance rates) were used as predictor variables. Furthermore, follow-up time was calculated as the time between start of the study (1 January 2000) and urinary culture and used as a potential confounder in the models.

### Analysis and statistical methods

Binary logistic regression was used to study the association between categorized use of antimicrobial drugs per group as defined above and trimethoprim resistance (no/yes). For each antimicrobial drug, we first calculated univariable ORs. Second, we performed a multivariable analysis for each antimicrobial drug group in which the OR was adjusted for the potential confounders age, sex, GFR, SES, diabetes, and follow-up time (model 1). Third, we performed an overall analysis of all antimicrobial drug groups in which the ORs of the antimicrobial drug groups were adjusted for all confounders and adjusted for the use of all other studied antimicrobial drug groups (model 2). For example, in model 2, the use of sulfonamides and trimethoprim was adjusted for age, sex, GFR, SES, diabetes, follow-up time, use of tetracylines, use of extended-spectrum penicillins, use of combinations of penicillins, incl. enzyme inhibitors, use of macrolides and lincosamides, use of fluoroquinolones, and use of nitrofurantoin during the study period. Furthermore, Spearman’s correlation coefficients were calculated for the use of all antimicrobial drug groups.

*p* < 0.05 was considered to be statistically significant. All statistical analyses were performed with IBM SPSS Statistics 24.

## Results

The study population consisted of 1264 individuals with urinary cultures positive for *E. coli* of whom 1011 (80.0%) were women, and with a median age of 75 years. The percentage resistance to trimethoprim of all *E. coli* isolates was 31.1%. Resistance percentages to other antimicrobials in trimethoprim-resistant *E. coli* were higher than in trimethoprim-sensitive *E. coli* isolates: 86.3% versus 26.5% were co-resistant to amoxicillin, 26.2% versus 9.2% to amoxicillin-clavulanic acid, 22.4% versus 4.7% to ciprofloxacin, and 13.0% versus 4.2% to nitrofurantoin (Table 1). Of all included individuals, 893 (70.6%) were prescribed at least one beta-lactam antimicrobial drug during the study period, 745 (58.9%) a sulfonamide and trimethoprim, 466 (39.9%) a fluoroquinolone, and 603 (47.7%) nitrofurantoin (Table 1).  Although resistance to all other antimicrobial drugs was higher in the trimethoprim-resistant *E. coli* isolates than in the trimethoprim-sensitive *E. coli* isolates, the overall use of antimicrobial drugs was not significantly higher in individuals with trimethoprim-resistant *E. coli* isolates. We also studied the correlations between the use of different antibiotic classes, which were all negligible to low and none of them was negative. For example, participants who had received a higher number of sulphonamides and trimethoprim prescriptions also had been prescribed a higher number of prescriptions of all other antimicrobial drug groups, but these correlations were low (Table 2).

**Table 1:** General characteristics of study population

	All participants (*n* = 1264)	Participants with UTI with trimethoprim-resistant *E. coli* (*n* = 393)
Age (years), median (IQR)	75.3 (66.5–83.3)	77.2 (67.6–85.1)
Women, *n* (%)	1011 (80.0)	323 (82.2)
Kidney function (GFR), median (IQR)	80.3 (69.8–90.1)	79.0 (68.8–89.2)
Diabetes, *n* (%)	170 (13.4)	58 (14.8)
Trimethoprim resistance, *n* (%)	393 (31.1)	393 (100)
Amoxicillin resistance, *n* (%)	570 (45.1)	339 (86.3)
Amoxicillin-clavulanic acid resistance, *n* (%)	183 (14.5)	103 (26.2)
Ciprofloxacin resistance, *n* (%)	129 (10.2)	88 (22.4)
Nitrofurantoin resistance, *n* (%)	88 (7.0)	51 (13.0)
Previous use of sulfonamides and trimethoprim, *n* (%)	745 (58.9)	264 (67.2)
Previous use of tetracyclines, *n* (%)	759 (60.0)	241 (61.3)
Previous use of extended-spectrum penicillins, *n* (%)	690 (54.6)	218 (55.4)
Previous use of combinations of penicillins, incl. enzyme inhibitors, *n* (%)	602 (47.6)	192 (48.9)
Previous use of macrolides and lincosamides, *n* (%)	568 (44.9)	184 (46.8)
Previous use of fluoroquinolones, *n* (%)	466 (39.9)	176 (44.8)
Previous use of nitrofurantoin, *n* (%)	603 (47.7)	188 (47.8)

General characteristics of the study population. The first column shows the characteristics for all participants, whereas the second column shows the characteristics only of participants who had a urinary tract infection caused by a trimethoprim-resistant *E. coli*. Kidney function is the glomerular filtration rate (GFR), according to the CKD-EPI equation. Furthermore, it shows the number (%) of individuals who had a UTI caused by *E*. *coli* resistant to several antimicrobial drugs and the number (%) of individuals who used at least one antimicrobial drug of different antimicrobial drug groups before culturing

**Table 2:** Spearman’s rank correlation coefficients for the use of antimicrobial drugs

	Sulfonamides and trimethoprim	Tetracyclines	Extended-spectrum penicillins	Combinations of penicillins with enzyme inhibitors	Macrolides and lincosamides	Fluoroquinolones	Nitrofurantoin
Sulfonamides and trimethoprim	–	0.19	0.09	0.15	0.14	0.40	0.39
Tetracyclines	0.19	–	0.30	0.25	0.38	0.21	0.19
Extended-spectrum penicillins	0.09	0.30	–	0.30	0.21	0.10	0.11
Combinations of penicillins with enzyme inhibitors	0.15	0.25	0.30	–	0.21	0.25	0.13
Macrolides and lincosamides	0.13	0.38	0.21	0.21	–	0.17	0.17
Fluoroquinolones	0.40	0.21	0.10	0.25	0.17	–	0.29
Nitrofurantoin	0.39	0.19	0.11	0.13	0.17	0.29	–

Spearman’s rank correlation coefficients for the correlations between the use of different antimicrobial drug groups. The Spearman’s rank correlation coefficient is used because of skewed distribution of the variables. The strength of the relation can be between − 1 (perfect negative correlation) to 1 (perfect positive correlation) with 0 meaning no correlation present. As a rule of thumb, the size of the strength of the correlation can be interpreted as follows: 0.9 to 1.0 (− 0.9 to − 1.0) very high correlation, 0.70 to 0.90 (− 0.70 to − 0.90) high correlation, 0.50 to 0.70 (− 0.50 to − 0.70) moderate correlation, 0.30 to 0.50 (− 0.30 to − 0.50) low correlation, and 0.0 to 0.30 (0.0 to − 0.30) negligible correlation [15]

Several antimicrobial drug classes, such as tetracyclines and fluoroquinolones, seemed to be associated with trimethoprim resistance in model 1, but this effect disappeared in model 2, in which antimicrobial drug use of one class was adjusted for potential confounding by use of other antimicrobial drug groups. In model 2, we showed that > 3 prescriptions of extended-spectrum penicillins were associated with trimethoprim resistance (OR 1.68; 95% CI 1.10–2.55), whereas combinations of penicillins incl. enzyme inhibitors were not. Also, > 3 prescriptions of a sulfonamide and trimethoprim were significantly associated with trimethoprim resistance (OR 2.22; 95% CI 1.51–3.26). In contrast, > 3 nitrofurantoin prescriptions were significantly associated with a lower frequency of resistance to trimethoprim (OR 0.60; 95% CI 0.39–0.92) (Table 3).

**Table 3:** Associations between previous use of antimicrobial drug groups and trimethoprim resistance

	No use	1 prescription	2 or 3 prescriptions	> 3 prescriptions
Sulfonamides and trimethoprim				
Number of cases (%)	129 (32.8)	80 (20.4)	75 (19.1)	109 (27.7)
Number of controls (%)	390 (44.8)	178 (20.4)	167 (19.2)	136 (15.6)
OR (95% CI) univariable	Ref	1.36 (0.98–1.89)	1.36 (0.97–1.90)	2.42 (1.76–3.34)*
OR (95% CI) in model 1	Ref	1.40 (1.00–1.95)*	1.32 (0.94–1.86)	2.28 (1.64–3.17)*
OR (95% CI) in model 2	Ref	1.45 (1.03–2.05)*	1.32 (0.92–1.89)	2.22 (1.51–3.26)*
Tetracyclines				
Number of cases (%)	152 (38.7)	84 (21.4)	61 (15.5)	96 (24.4)
Number of controls (%)	353 (40.5)	178 (20.4)	178 (20.4)	162 (18.6)
OR (95% CI) univariable	Ref	1.10 (0.79–1.51)	0.80 (0.56–1.13)	1.38 (1.00–1.89)*
OR (95% CI) in model 1	Ref	1.10 (0.79–1.52)	0.84 (0.59–1.19)	1.43 (1.03–1.97)*
OR (95% CI) in model 2	Ref	0.98 (0.69–1.37)	0.72 (0.50–1.05)	1.11 (0.76–1.61)
Extended-spectrum penicillins				
Number of cases (%)	175 (44.5)	83 (21.1)	72 (18.3)	63 (16.0)
Number of controls (%)	399 (45.8)	216 (24.8)	164 (18.8)	92 (10.6)
OR (95% CI) univariable	Ref	0.88 (0.64–1.19)	1.00 (0.72–1.39)	1.56 (1.08–2.25)*
OR (95% CI) in model 1	Ref	0.93 (0.68–1.27)	1.07 (0.77–1.50)	1.80 (1.23–2.63)*
OR (95% CI) in model 2	Ref	0.93 (0.67–1.28)	1.04 (0.73–1.48)	1.68 (1.10–2.55)*
Combinations of penicillins, incl. enzyme inhibitors				
Number of cases (%)	201 (51.1)	84 (21.4)	62 (15.8)	46 (11.7)
Number of controls (%)	461 (52.9)	186 (21.4)	142 (16.3)	82 (9.4)
OR (95% CI) univariable	Ref	1.03 (0.76–1.41)	1.00 (0.71–1.41)	1.29 (0.87–1.91)
OR (95% CI) in model 1	Ref	1.06 (0.78–1.45)	1.09 (0.77–1.54)	1.39 (0.92–2.08)
OR (95% CI) in model 2	Ref	0.98 (0.71–1.36)	0.91 (0.63–1.32)	0.98 (0.62–1.55)
Macrolides and lincosamides				
Number of cases (%)	209 (53.2)	88 (22.4)	55 (14.0)	41 (10.4)
Number of controls (%)	487 (55.9)	168 (19.3)	132 (15.2)	84 (9.6)
OR (95% CI) univariable	Ref	1.22 (0.90–1.66)	0.97 (0.68–1.38)	1.14 (0.76–1.71)
OR (95% CI) in model 1	Ref	1.31 (0.96–1.79)	1.06 (0.74–1.53)	1.27 (0.83–1.91)
OR (95% CI) in model 2	Ref	1.21 (0.87–1.67)	0.90 (0.61–1.32)	0.97 (0.61–1.54)
Fluoroquinolones				
Number of cases (%)	217 (55.2)	63 (16.0)	58 (14.8)	55 (14.0)
Number of controls (%)	581 (66.7)	118 (13.5)	95 (10.9)	77 (8.8)
OR (95% CI) univariable	Ref	1.43 (1.01–2.02)*	1.64 (1.14–2.35)*	1.91 (1.31–2.80)*
OR (95% CI) in model 1	Ref	1.45 (1.02–2.06)*	1.59 (1.10–2.29)*	1.89 (1.28–2.79)*
OR (95% CI) in model 2	Ref	1.33 (0.92–1.93)	1.43 (0.96–2.13)	1.51 (0.96–2.37)
Nitrofurantoin				
Number of cases (%)	205 (52.2)	64 (16.3)	69 (17.6)	55 (14.0)
Number of controls (%)	456 (52.4)	163 (18.7)	130 (14.9)	122 (14.0)
OR (95% CI) univariable	Ref	0.87 (0.63–1.22)	1.18 (0.84–1.65)	1.00 (0.70–1.44)
OR (95% CI) in model 1	Ref	0.85 (0.60–1.19)	1.11 (0.79–1.57)	0.99 (0.68–1.44)
OR (95% CI) in model 2	Ref	0.72 (0.51–1.04)	0.86 (0.59–1.27)	0.60 (0.39–0.92)*

Associations between the previously prescribed number (1, 2 or 3, > 3 compared with none) of prescriptions of antimicrobial agents for individuals with a UTI caused by an *E. coli* resistant to trimethoprim (cases) compared with individuals with a UTI caused by an *E. coli* susceptible to trimethoprim (controls). For each antimicrobial drug group, it shows the univariable OR; the OR adjusted for the possible confounders sex, age, diabetes, GFR, SES, and follow-up time (model 1) and the OR adjusted for sex, age, diabetes, GFR, SES, and follow-up time; and the use of other antimicrobial drug group prescriptions (model 2) *significant with p < 0.05

The time period since the last prescription of extended-spectrum penicillins (1–3 months; OR 2.86; 95% CI 1.29–6.34) was associated with trimethoprim resistance in model 2. This association was not seen for combinations of penicillins and enzyme inhibitors. Furthermore, an association was demonstrated between the use of sulfonamides and trimethoprim in the 1–3 months before culture (OR 2.22; 95% CI 1.27–3.87) and trimethoprim resistance. Although there was no association with the use of fluoroquinolones 1–3 months before culture, there was one for 3–12 months (OR 2.28; 95% CI 1.33–3.19). No association was found between the use of nitrofurantoin and trimethoprim resistance in this model 2 using the time intervals (Table 4). However, when adjusting the timing of the last nitrofurantoin prescription for the number of prescriptions of the other antimicrobial drug groups instead of the timing of the last prescription of these groups, nitrofurantoin was associated with less trimethoprim resistance (1–3 months: OR 0.50; 95% CI 0.27–0.90).

**Table 4:** Associations between the timing of the last prescription of several antimicrobial drug groups and trimethoprim resistance

	No use	> 12 months	3–12 months	1–3 months
Sulfonamides and trimethoprim				
Number of cases (%)	110 (28.0)	101 (25.7)	37 (9.4)	25 (6.4)
Number of controls (%)	377 (43.3)	327 (37.5)	73 (8.4)	39 (4.5)
OR (95% CI) univariable	Ref	1.06 (0.78–1.44)	1.74 (1.11–2.72)*	2.20 (1.27–3.79)*
OR (95% CI) in model 1	Ref	1.04 (0.76–1.42)	1.71 (1.08–2.70)*	2.23 (1.28–3.88)*
OR (95% CI) in model 2	Ref	1.00 (0.72–1.39)	1.66 (1.00–2.77)*	2.22 (1.27–3.87)*
OR (95% CI) in model 2 adjusted for number of prescriptions	Ref	1.01 (0.72–1.42)	1.55 (0.95–2.52)	2.24 (1.25–4.02)*
Tetracyclines				
Number of cases (%)	152 (38.7)	197 (50.1)	25 (6.4)	12 (3.1)
Number of controls (%)	352 (40.4)	437 (50.2)	62 (7.1)	16 (1.8)
OR (95% CI) univariable	Ref	1.04 (0.81–1.35)	0.93 (0.57–1.54)	1.74 (0.80–3.76)
OR (95% CI) in model 1	Ref	1.07 (0.82–1.39)	0.92 (0.56–1.52)	1.80 (0.83–3.93)
OR (95% CI) in model 2	Ref	0.90 (0.66–1.21)	0.75 (0.43–1.32)	1.50 (0.64–3.53)
OR (95% CI) in model 2 adjusted for number of prescriptions	Ref	0.89 (0.66–1.18)	0.79 (0.48–1.29)	1.52 (0.68–3.39)
Extended-spectrum penicillins				
Number of cases (%)	174 (44.3)	166 (42.2)	20 (5.1)	16 (4.1)
Number of controls (%)	393 (45.1)	379 (43.5)	52 (6.0)	15 (1.7)
OR (95% CI) univariable	Ref	0.99 (0.77–1.28)	0.87 (0.50–1.50)	2.41 (1.17–4.98)*
OR (95% CI) in model 1	Ref	1.06 (0.81–1.37)	0.97 (0.56–1.69)	2.74 (1.31–5.73)*
OR (95% CI) in model 2	Ref	1.01 (0.75–1.36)	0.95 (0.52–1.74)	2.86 (1.29–6.34)*
OR (95% CI) in model 2 adjusted for number of prescriptions	Ref	1.00 (0.76–1.32)	0.87 (0.49–1.56)	2.91 (1.36–6.23)*
Combinations of penicillins, incl. enzyme inhibitors				
Number of cases (%)	186 (47.3)	128 (32.6)	31 (7.9)	16 (4.1)
Number of controls (%)	432 (49.6)	277 (31.8)	57 (6.5)	28 (3.2)
OR (95% CI) univariable	Ref	1.07 (0.82–1.41)	1.26 (0.79–2.02)	1.33 (0.70–2.51)
OR (95% CI) in model 1	Ref	1.16 (0.88–1.53)	1.24 (0.77–1.99)	1.31 (0.68–2.50)
OR (95% CI) in model 2	Ref	1.11 (0.81–1.52)	0.96 (0.73–1.28)	1.26 (0.62–2.57)
OR (95% CI) in model 2 adjusted for number of prescriptions	Ref	1.00 (0.75–1.35)	1.00 (0.62–1.61)	1.07 (0.54–2.12)
Macrolides and lincosamides				
Number of cases (%)	208 (52.9)	148 (37.7)	22 (5.6)	9 (2.3)
Number of controls (%)	484 (55.6)	311 (35.7)	50 (5.7)	15 (1.7)
OR (95% CI) univariable	Ref	1.11 (0.86–1.43)	1.02 (0.60–1.73)	1.40 (0.60–3.24)
OR (95% CI) in model 1	Ref	1.21 (0.93–1.58)	1.13 (0.66–1.92)	1.43 (0.60–3.37)
OR (95% CI) in model 2	Ref	1.07 (0.80–1.42)	0.90 (0.51–1.60)	1.35 (0.55–3.29)
OR (95% CI) in model 2 adjusted for number of prescriptions	Ref			
Fluoroquinolones				
Number of cases (%)	209 (53.2)	97 (24.7)	33 (8.4)	19 (4.8)
Number of controls (%)	559 (64.2)	193 (22.2)	37 (4.2)	25 (2.9)
OR (95% CI) univariable	Ref	1.34 (1.01–1.80)*	2.39 (1.45–3.92)*	2.03 (1.09–3.77)*
OR (95% CI) in model 1	Ref	1.34 (0.99–1.80)	2.50 (1.51–4.15)*	1.86 (0.99–3.48)
OR (95% CI) in model 2	Ref	1.19 (0.84–1.67)	2.02 (1.14–3.65)*	1.66 (0.83–3.34)
OR (95% CI) in model 2 adjusted for number of prescriptions	Ref	1.13 (0.81–1.58)	2.28 (1.33–3.19)*	1.59 (0.82–3.07)
Nitrofurantoin				
Number of cases (%)	180 (45.8)	91 (23.2)	39 (9.9)	17 (4.3)
Number of controls (%)	386 (44.3)	183 (21.0)	64 (7.3)	57 (6.5)
OR (95% CI) univariable	Ref	1.07 (0.78–1.45)	1.31 (0.85–2.02)	0.64 (0.36–1.13)
OR (95% CI) in model 1	Ref	0.99 (0.72–1.37)	1.27 (0.84–1.92)	0.64 (0.36–1.12)
OR (95% CI) in model 2	Ref	0.82 (0.57–1.19)	0.91 (0.57–1.47)	0.54 (0.29–1.01)
OR (95% CI) in model 2 adjusted for number of prescriptions	Ref	0.76 (0.54–1.08)	0.97 (0.61–1.53)	0.50 (0.27–0.90)*

Associations between the timing (1–3 months, 3–12 months, > 12 months before culture compared with no use) of the last prescription of antimicrobial agents for individuals with a UTI caused by an *E. coli* resistant to trimethoprim (cases) compared with individuals with a UTI caused by an *E. coli* susceptible for trimethoprim (controls). For each antimicrobial drug group, it shows the univariable OR, the OR adjusted for the possible confounders sex, age, diabetes, GFR, SES, and follow-up time (model 1) and the OR adjusted for the sex, age, diabetes, GFR, SES, and follow-up time; and antimicrobial drug group prescriptions of the other antimicrobial drug groups (model 2) Furthermore, it shows model 2 but adjusted for the number of prescriptions of the other antimicrobial drug groups instead of the variable that describes the timing of these prescriptions. *significant with p < 0.05

## Discussion

In this study, we showed that trimethoprim use and extended-spectrum penicillins use (such as amoxicillin) were significantly associated with trimethoprim resistance in *E. coli* causing UTIs, both for the number of prescriptions (> 3 prescriptions) as well as for the time interval between the last prescription and culture (prescription 1–3 months before culture). Additionally, the use of > 3 prescriptions of nitrofurantoin was associated with a lower frequency of trimethoprim resistance on a patient level. Both these results confirm at an individual patient level the findings from the association study at population level, mentioned in the “Introduction” section [5].

Nitrofurantoin use, as assessed by prescriptions, was associated with a lower frequency of trimethoprim resistance, but only after adjustment for the number of other antimicrobial drug prescriptions, including trimethoprim. This suggests that nitrofurantoin use is inversely associated with trimethoprim resistance in individuals with a high frequency of antimicrobial drug use, possibly due to recurrent UTIs. This result was not found in the model that studied the timing of the last prescription, possibly because participants who were prescribed nitrofurantoin shortly before the urine culture were not or less frequently prescribed another antimicrobial drug for a UTI episode. Co-resistance to trimethoprim and nitrofurantoin was low (13%), but higher than overall resistance to nitrofurantoin (7%) and there was a low but positive correlation between sulfonamides use and trimethoprim or nitrofurantoin use. Thus, lower trimethoprim resistance might not to be caused by a decreased frequency of trimethoprim use, but it may be hypothesized that trimethoprim-resistant *E. coli* are eradicated by nitrofurantoin.

Despite the high frequency of use, resistance to nitrofurantoin remains rather low in contrast to other antimicrobial drugs [11]. This might be explained by the observation that *E. coli* mutants resistant to nitrofurantoin were less able to multiply, which is a disadvantage compared with nitrofurantoin-sensitive *E. coli* [16]. Furthermore, the nfsA and nfsB, genes, which play a role in nitrofurantoin resistance are chromosomal and not plasmid-mediated, diminishing the chance of transfer of these genes to other bacteria. However, recently, the plasmid-mediated *oqxAB* has also been shown to play a role in nitrofurantoin resistance [17]. These characteristics of nitrofurantoin make it an antimicrobial drug of high interest in this era of antimicrobial resistance.

The association between sulfonamides and trimethoprim use and trimethoprim resistance was expected and has been described before [6, 18]. We also confirm at the patient level the association between extended-spectrum penicillins use, such as amoxicillin, and trimethoprim resistance [8]. Furthermore, the correlation between sulfonamides and trimethoprim use and extended-spectrum penicillins use is low. Moreover, since we adjusted for trimethoprim use in the model, it seems unlikely that the association between amoxicillin use and trimethoprim resistance is the result of a combination of a high number of prescriptions of amoxicillin and trimethoprim. Our data suggest that co-resistance plays an important role, especially in individuals with high antimicrobial drug use.

The association between fluoroquinolones use and trimethoprim resistance remains unclear. In our analyses, the association between fluoroquinolone use and trimethoprim resistance disappeared after adjustment for other antimicrobial drug groups. In the time interval model, the association with the last prescription 3–12 months before culture could not be confirmed in other time periods. The association between fluoroquinolones use and trimethoprim resistance should therefore be investigated further. Also, we did not find any associations with macrolides and lincosamides use, although Pouwels et al. did find a protective effect of macrolide use on the resistance to trimethoprim [8]. This is possibly explained by differences in prescribing patterns between the Netherlands and UK.

A strength of this study is that we confirmed the results of an earlier association study with aggregated general practice data [5] using a nested case-control design with individual patient data. On the contrary, a limitation of the study is the fact that we use filled prescription data for antimicrobial drugs but do not know whether patients were adherent to pharmacotherapy. However, since individuals with infections visit their GP because of complaints and antibiotics are usually seen by patients as safe and effective, it is reasonable to assume that patients actually have taken the drugs. Furthermore, our results could be influenced by residual confounding. For example, it cannot be excluded that there are differences between GPs in prescribing antimicrobial drugs and in sending cultures, although there is a national guideline that give recommendations for diagnostics and treatment of UTIs by GPs [10]. Finally, the design of the study did not allow us to confirm our results by studying the genetic associations of resistance genes in these *E. coli* isolates.

In conclusion, in individual patients, the use of extended-spectrum penicillins, such as amoxicillin, is associated with trimethoprim resistance, possibly via selection by co-resistance. Importantly, use of nitrofurantoin is associated with lower trimethoprim resistance. This indicates that co-resistance could be important to take into account when prescribing antimicrobial drugs and that resistance testing is important in individuals with recurrent UTIs.

## Electronic supplementary material

ESM 1 (DOCX 15 kb)

## Data Availability

The datasets analyzed during the current study are not publicly available due to privacy agreements of the participants of the study but are available from the corresponding author on reasonable request.
