# Supplementary material for: Use of other antimicrobial drugs is associated with trimethoprim resistance in patients with urinary tract infections caused by E. coli
Source: Eur J Clin Microbiol Infect Dis. 2019 Sep 7;38(12):2283–90. doi: 10.1007/s10096-019-03672-2 (PMC6858404; doi:10.1007/s10096-019-03672-2)
Supplement: Supplementary file 1 — (DOCX 15 kb) [file 10096_2019_3672_MOESM1_ESM.docx]

**Supplementary table 1: Spearman’s rank correlation coefficients for the use of antimicrobial drug groups**

|  | Sulfonamides and trimethoprim | Tetracyclines | Penicillins with extended spectrum | Combinations of penicillins with enzyme inhibitors | Macrolides and lincosamides | Fluoroquinolones | Nitrofurantoin |
| --- | --- | --- | --- | --- | --- | --- | --- |
| Sulfonamides and trimethoprim | - | 0.19 | 0.09 | 0.15 | 0.14 | 0.40 | 0.39 |
| Tetracyclines | 0.19 | - | 0.30 | 0.25 | 0.38 | 0.21 | 0.19 |
| Penicillins with extended spectrum | 0.09 | 0.30 | - | 0.30 | 0.21 | 0.10 | 0.11 |
| Combinations of penicillins with enzyme inhibitors | 0.15 | 0.25 | 0.30 | - | 0.21 | 0.25 | 0.13 |
| Macrolides and lincosamides | 0.13 | 0.38 | 0.21 | 0.21 | - | 0.17 | 0.17 |
| Fluoroquinolones | 0.40 | 0.21 | 0.10 | 0.25 | 0.17 | - | 0.29 |
| Nitrofurantoin | 0.39 | 0.19 | 0.11 | 0.13 | 0.17 | 0.29 | - |

Spearman’s rank correlation coefficients for the correlations between the use of different antimicrobial drug groups. The Spearman’s rank correlation coefficient is used because of skewed distribution of the variables. The strength of the relation can be between -1 (perfect negative correlation) to 1 (perfect positive correlation) with 0 meaning no correlation present. As a rule of thumb, the size of the strength of the correlation can be interpreted as follows: 0.9 to 1.0 (-0.9 to -1.0) very high correlation; 0.70 to 0.90 (-0.70 to -0.90) high correlation; 0.50 to 0.70 (-0.50 to -0.70) moderate correlation; 0.30 to 0.50 (-0.30 to -0.50) low correlation and 0.0 to 0.30 (0.0 to -0.30) negligible correlation.[17]
